# Salvage high-dose rate brachytherapy for myxofibrosarcoma of the brachium: a technical report

**DOI:** 10.1093/jrr/rrad041

**Published:** 2023-06-10

**Authors:** Naoya Murakami, Fumihiko Nakatani, Kana Takahashi, Satoshi Nakamura, Hiroshi Igaki, Naoto Shikama

**Affiliations:** Department of Radiation Oncology, Juntendo University School of Medicine, 3-1-3, Bunkyo-ku, Tokyo 113-8431, Japan; Department of Radiation Oncology, National Cancer Center Hospital, 5-1-1, Chuo-ku, Tokyo 104-0045, Japan; Department of Musculoskeletal Oncology and Rehabilitation, National Cancer Center Hospital East, 6-5-1, Kashiwa-shi, Chiba 277-8577, Japan; Department of Radiation Oncology, Juntendo University School of Medicine, 3-1-3, Bunkyo-ku, Tokyo 113-8431, Japan; Division of Radiation Safety and Quality Assurance, National Cancer Center Hospital, 5-1-1, Chuo-ku, Tokyo 104-0045, Japan; Department of Radiation Oncology, Juntendo University School of Medicine, 3-1-3, Bunkyo-ku, Tokyo 113-8431, Japan; Department of Radiation Oncology, National Cancer Center Hospital, 5-1-1, Chuo-ku, Tokyo 104-0045, Japan

## Abstract

An 80-year-old male presented with T1N0M0 myxofibrosarcoma in or next to the humeral canal, which is located between the biceps and triceps of the right upper arm. Because the tumor was close to critical anatomical structures such as the brachial artery, median nerve and ulnar nerve, it was deemed impossible to perform limb-sparing surgery with an adequate resection margin. Therefore, preoperative external beam radiation therapy (EBRT) followed by limb-sparing surgery was offered. Magnetic resonance imaging taken after 40 Gy/20 fractions of EBRT showed an inadequate response, and limb-sparing surgery was not deemed possible at this point. Amputation of the right arm was offered, but the patient refused. Therefore, salvage high-dose-rate interstitial brachytherapy (HDR-ISBT) was offered. Under local anesthesia and sedation, 14 plastic needles were inserted, and 36 Gy in 6 fractions of HDR-ISBT was performed. Although radiation-induced incomplete paralysis of the median nerve was noted, no local progression or distant metastasis was found on the CT that was taken 2 years after the treatment.

## INTRODUCTION

Soft tissue sarcoma (STS) is one of the rare mesenchymal malignancies, accounting for <1% of all adult cancers in Japan. The standard treatment for STS is surgery with or without adjuvant radiotherapy (RT) [1–3]. Myxofibrosarcoma (MFS) is one of the STSs affecting mostly elderly patients, and the standard management of MFS is the same as that of STSs with a propensity for local recurrence [4]. Since the evolution of the surgical technique, limb-sparing surgery plus perioperative RT has provided equivalent local control and superior functional outcomes compared with amputation for STSs arising from extremities [5]. However, when limb-sparing surgery is not feasible because of the proximity of a feeding artery or major nerve, amputation still has an important role. As mentioned earlier, because MFS affects elderly patients, sometimes it is difficult for them to endure a large surgery or they refuse to accept loss of function after amputation. In such a case, RT can be offered as an alternative treatment modality. Usually, external beam radiation therapy (EBRT) is selected in this situation, but due to the tolerance dose for normal tissues such as skin, bone, artery or subcutaneous tissues, 60–70 Gy would be the upper limit that can be delivered by EBRT alone, and because STSs are known to be radioresistant tumors, controlling the disease only by EBRT is generally difficult.

Here, we report a case of MFS in the right upper arm. Initially, preoperative RT followed by limb-sparing surgery was planned, but an interscapulothoracic amputation was required due to the inadequate response to the RT, which was subsequently salvaged by interstitial brachytherapy.

## CASE PRESENTATION

**Fig. 1:**
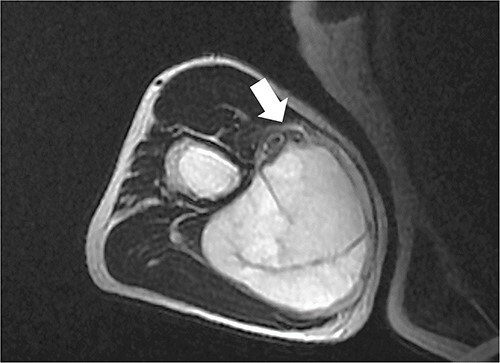
T2-weighted axial magnetic resonance imaging of the right upper arm. High-intensity mass can be seen between the biceps and triceps. The white arrow indicates the neurovascular bundle.

An 80-year-old male with hypertension and diabetes as comorbidities presented with an upper arm subcutaneous mass to the previous hospital. Histopathology proved that it was a MFS, and because the maximal tumor size was 4.5 cm without any regional lymph node or distant metastasis, it was classified as T1N0M0 Stage I. This tumor was found within or near the humeral canal, which is located between the biceps and triceps of the right upper arm (Fig. 1). Due to the proximity of the tumor to critical anatomical structures such as the brachial artery, median nerve and ulnar nerve, limb-sparing surgery with an adequate resection margin was deemed impossible. Therefore, preoperative EBRT followed by limb-sparing surgery was offered. Preoperative radiation therapy of 40 Gy in 20 fractions was delivered in the previous hospital, where the clinical target volume (CTV) was set with a 1.5 cm margin around the gross target volume (GTV). After EBRT, magnetic resonance imaging was taken to assess the response. Unfortunately, the initially expected response was not obtained, and limb-sparing surgery was not deemed possible at this point. Interscapulothoracic amputation of the right arm was offered, but the patient refused. Because further EBRT was deemed impossible at the previous hospital, he was referred to our hospital to seek the possibility of salvage high-dose-rate interstitial brachytherapy (HDR-ISBT). Because there was no space between the tumor and neurovascular bundle, it was explained that there was a high risk of radiation therapy (RT)-induced late neurotoxicity, but he chose to undergo treatment nonetheless.

## INTERSTITIAL BRACHYTHERAPY

**Fig. 2:**
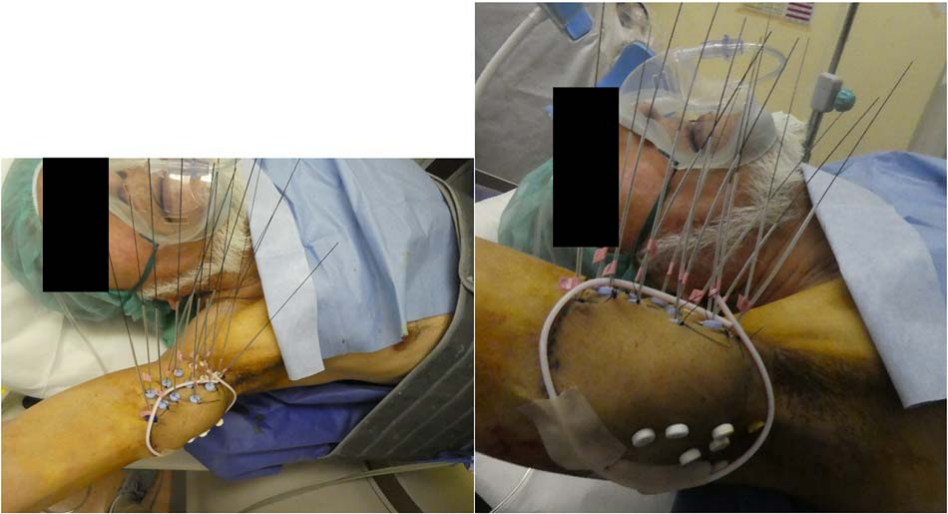
Under axillary brachial plexus blockade, evenly spaced 14 plastic interstitial needles were inserted parallelly guided by ultrasound so as not to injure the brachial artery. The needles were fixed by buttons attached to the needles and sutured to the skin. To aid the CTV contouring on the planning CT, which was set adding 1.5 cm around the GTV, a radiopaque marker was placed on the skin.

**Fig. 3:**
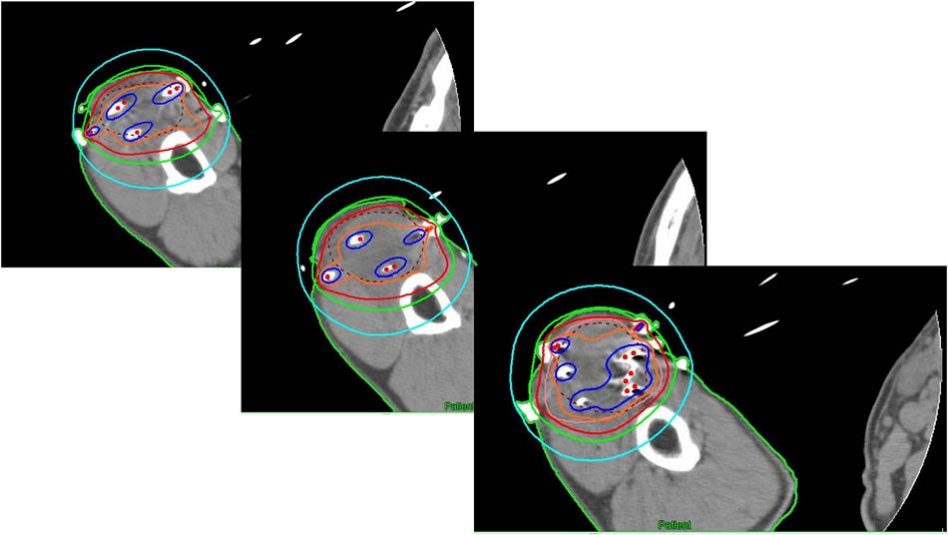
The dose distribution of HDR-ISBT of the right upper arm on the axial image of the planning CT. The red, orange and blue lines represent 100, 150 and 200% isodose lines, respectively. The red dotted line represents the gross tumor volume at the time of brachytherapy.

**Fig. 4:**
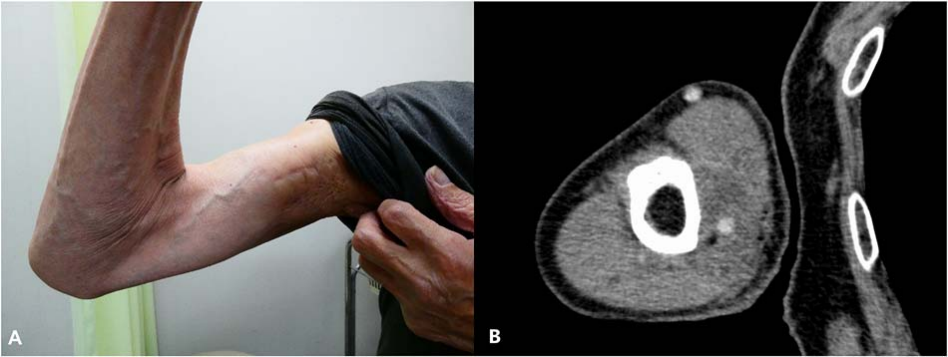
(**A**) The right upper arm. Mild pigmentation and atrophy of the irradiated site are shown. (**B**) An axial image of the contrast enhanced CT that was taken 2 years after the treatment. No local progression was noted. The circulation of the brachial artery can be seen.

The interval between the last day of EBRT and the first day of HDR-ISBT was 26 days. Under axillary brachial plexus blockade [6] performed proximal to the tumor guided by ultrasound, local anesthesia and sedation, evenly spaced 14 plastic interstitial needles were inserted parallelly guided by ultrasound so as not to injure the brachial artery. Similar to the technique used in breast interstitial brachytherapy, trocars were initially inserted, and subsequently they were replaced with plastic catheters with fixation bottoms. Needle insertion was initiated from the deepest plane to avoid any potential interference with the ultrasound image that may result from the artifacts caused by superficial plane needles. In cases where the obstacle of the humerus impeded obtaining a 1.5 cm margin from the GTV, this objective was not always feasible. Finally, the needles were fixed by buttons attached to the needles and sutured to the skin (Fig. 2). These 14 needles were intended to cover the CTV, which was set with a 1.5 cm margin around the GTV. After the needle insertion, a planning computed tomography (CT) was taken with a 2 mm thickness by a large-bore CT scanner located in the brachytherapy suite (Aquilion® LB, Canon, Tokyo, Japan). Dose calculation was performed with the brachytherapy planning system (Oncentra®, Elekta, Veenendaal, The Netherlands) so that the 6 Gy isodose line covered the CTV (Fig. 3). The CTV D_90_ and V_100_ were 7.67 Gy and 99.6%, whereas the GTV D_90_ and V_100_ were 8.62 Gy and 100%, respectively. Because the median nerve could not be identified on CT images, the median nerve dose could not be evaluated. The needle insertion was performed once, and irradiation was conducted b.i.d. at a 6-h interval. A total of 36 Gy in six fractions of HDR-ISBT was delivered. To mitigate the risk of iatrogenic implantation, the entire needle track was irradiated up to the skin only during the first fraction of brachytherapy. No severe acute adverse events related to needle insertion or removal were noted. Although mild pigmentation, atrophy and hardening of the subcutaneous tissue of the irradiated site and radiation-induced incomplete paralysis of the median nerve were noted, such that he cannot use chopsticks to eat but still can use a spoon, no local progression or distant metastasis was found on the CT that was taken 2 years after the treatment (Fig. 4). Concerning the size change of the tumor, although it was 4.5 × 4 cm prior to treatment, it turned 4 × 3 cm 5 months after HDR-ISBT, then 3 × 2 cm 11 months after HDR-ISBT and was difficult to measure 2 years after the treatment without any enhanced lesions, as shown in Fig. 4. Unfortunately, 3 months after HDR-ISBT, he complained of mild difficulty using the chopsticks due to incomplete paralysis, which eventually progressed to grade 3 incomplete paralysis over time.

## DISCUSSION

The standard treatment for STS is surgery with or without adjuvant RT [1–3], and MFS is also mainly managed with primary surgery followed by radiation therapy when needed [4]. Along with EBRT, brachytherapy can be used perioperatively [7, 8]. In addition, the use of brachytherapy is also considered in cases of unresectable, recurrent or metastatic disease in the American Society for Radiation Oncology guidelines [3]. A previous article reported the usefulness of brachytherapy in controlling metastatic extra-skeletal myxoid chondrosarcoma [9].

Carbon ion RT demonstrated favorable local control for unresectable STS [10] and can be an option for unresectable STS; however, because the late radiation-induced bone fracture is a serious problem after carbon ion radiotherapy for the STS in the extremities and only a limited number of institutions can offer carbon ion radiotherapy, it was not selected for the patient described in this article.

It has been reported that MFS tends to progress locally, and a positive or close margin in the resected specimen predicts local recurrence [4]. Therefore, it might be possible that 2-year local control achieved in this case was attributable to an adequate margin added around GTV. In the case reported in this article, only 1.5 cm of margin around the GTV was adopted, whereas the American Brachytherapy Society (ABS) consensus statement advocates for margins of at least 2 cm craniocaudally and 1–2 cm radially [11]. It is important to note that the ABS consensus statement pertains to the CTV margin in postoperative settings, which may necessitate wider margins in comparison with primary settings due to the imprecision stemming from the primary tumor removal and postoperative tissue changes. Considering the 40 Gy of EBRT that this case already underwent, a reduced CTV margin of 1.5 cm for the boost brachytherapy, as implemented in this case, may prove to be adequate. Nonetheless, it is crucial to further investigate the actual CTV margin in the primary settings following the preceding EBRT, which should be investigated further in future research endeavors, as the successful local control noted in this case may have merely been serendipitous.

In this case, despite the time gap of almost a month between the preceding EBRT and brachytherapy, we deemed that the boost dose was sufficient for this case. In the management of uterine cervical cancer, four times 6 Gy of brachytherapy can be applied following 40 Gy of EBRT. However, we perceived STS to be more radioresistant compared with cervical cancer and hence concluded that four times of 6 Gy would be inadequate. As a result, we finally chose six times of 6 Gy in this case as a brachytherapy boost. The appropriate dose schedule in the primary settings will also be established in future studies.

Because brachytherapy can safely deliver a conformal and high dose while avoiding surrounding normal tissues, it can deliver higher doses to the target volume than EBRT can; therefore, when combined with EBRT, better local control would be expected. In the current case presented in this article, assuming that the α/β value for MFS is 10 Gy and that the linear quadrant model could be applied, a total of 106.9 Gy has been delivered combining EBRT and brachytherapy doses, which would have been impossible to deliver by EBRT alone. Even so, because there are only limited reports regarding primary radiation therapy involving brachytherapy for MFS and the time interval between EBRT and brachytherapy was as long as 26 days, frankly speaking, we have not expected such a favorable local control with salvage HDR-ISBT, and the current result was a big surprise for us. As written in the case presentation, since the tumor exhibited a slow response to brachytherapy, a further follow-up period may be necessary to confirm its complete remission. Nonetheless, the authors believe that for patients who cannot be operated on but can be treated with brachytherapy, it should be considered as an alternative treatment modality. Of course, when limb-sparing surgery is possible, the functional outcome for limb-sparing surgery followed by postoperative RT is much better because, even with brachytherapy, it is not possible to deliver a tumoricidal dose while sparing the medial nerve under 50 Gy, which is dose tolerance for peripheral nerve when the median nerve is next to the tumor. However, when limb-sparing surgery is not possible, EBRT followed by a brachytherapy boost can be considered as an alternative treatment. Because of the paucity of reports on brachytherapy for STSs, there are issues that should be clarified by future studies: (i) appropriate dose combination of EBRT and brachytherapy boost; (ii) appropriate time gap between EBRT and brachytherapy; (iii) appropriate CTV margin around GTV for brachytherapy; and (iv) dose constraints for surrounding tissues such as skin, subcutaneous tissues, arteries and bone. Still, the authors believe that brachytherapy boost after EBRT for STSs can be a promising treatment that should be further investigated in future studies.

## CONFLICT OF INTEREST

H.I. receives a research fund from Elekta KK outside the submitted work. The other authors have no conflict of interests to declare.

## FUNDING

None.

## DATA AVAILABILITY

The data underlying this article will be shared on reasonable request to the corresponding author.
